# Periprosthetic infection: where do we stand with regard to Gram stain?

**DOI:** 10.1080/17453670902804943

**Published:** 2009-02-01

**Authors:** Elie Ghanem, Constantinos Ketonis, Camilo Restrepo, Ashish Joshi, Robert Barrack, Javad Parvizi

**Affiliations:** ^1^Department of Orthopaedic Surgery, Thomas Jefferson University Hospital, Rothman InstitutePhiladelphia, PAUSA; ^2^The Barnes Jewish HospitalSt Louis, MOUSA

## Abstract

**Background and purpose** One of the routinely used intraoperative tests for diagnosis of periprosthetic infection (PPI) is the Gram stain. It is not known if the result of this test can vary according to the type of joint affected or the number of specimen samples collected. We examined the role of this diagnostic test in a large cohort of patients from a single institution.

**Materials and methods** A positive gram stain was defined as the visualization of bacterial cells or “many neutrophils” (> 5 per high-power field) in the smear. The sensitivity, specificity, and predictive values of each individual diagnostic arm of Gram stain were determined. Combinations were performed in series, which required both tests to be positive to confirm infection, and also in parallel, which necessitated both tests to be negative to rule out infection.

**Results** The presence of organisms and “many” neutrophils on a Gram smear had high specificity (98–100%) and positive predictive value (89–100%) in both THA and TKA. The sensitivities (30–50%) and negative predictive values (70–79%) of the 2 tests were low for both joint types. When the 2 tests were combined in series, the specificity and positive predictive value were absolute (100%). The sensitivity and the negative predictive value improved for both THA and TKA (43–64% and 82%, respectively).

**Interpretation** Although the 2 diagnostic arms of Gram staining can be combined to achieve improved negative predictive value (82%), Gram stain continues to have little value in ruling out PPI. With the advances in the field of molecular biology, novel diagnostic modalities need to be designed that can replace these traditional and poor tests.

## Introduction

There are numerous diagnostic tools to differentiate between septic and aseptic failure of a total joint arthroplasty including serology, joint fluid analysis and culture, and radionuclide imaging (Levitsky et al. 1991, Barrack et al. 1997, Spangehl et al. 1999a, Reinartz et al. 2005, Parvizi et al. 2006). The preoperative tests are not absolute, however, and some patients will require further intraoperative investigation.

The intraoperative tests that are available include frozen sections, histological evaluation of specimens, or performing Gram staining—which can detect the presence of organisms and neutrophils in tissue and fluid samples. Frozen section has been thoroughly investigated, and a general consensus regarding its usefulness has been reached (Feldman et al. 1995, Pandey et al. 2000). Few studies have examined in depth the diagnostic value of Gram stain in diagnosing PPI after total joint arthroplasty (Atkins et al. 1998, Spangehl et al. 1999b). No one has yet compared the efficacy of Gram stain in hip (THA) and knee (TKA) arthroplasty or explored the possibility of using the presence of “many” stained neutrophils as criteria for diagnosing PPI in a large cohort of patients. Combining the two arms of Gram staining (the presence of stained organisms or of “many” neutrophils) may improve the sensitivity and negative predictive value that this test lacks. We also hypothesized that increasing the number of intraoperative samples can improve the diagnostic value of Gram stain.

## Patients and methods

We performed a review of the hip and knee arthroplasty registry at our institution over a 5-year period (2001–2005). Institutional review board approval was obtained. Patients who underwent revision arthroplasty for an aseptic etiology or infection were included on condition that tissue and fluid cultures were obtained intraoperatively and Gram staining was performed on those samples. The joint arthroplasty was considered to be infected if an organism could be isolated from either the preoperative or intraoperative cultures on solid media. If culture failed to indicate an organism, then PPI was diagnosed if both the leukocyte count was > 1,760 cells/µL and polymorphonuclear cell count was > 73% (Parvizi et al. 2006), or if there was either a draining sinus tract or an abscess present. A total of 704 joint arthroplasties (419 THA, 285 TKA) underwent revision surgery for aseptic reasons, while 299 arthroplasties were revised for deep infection (132 THA, 168 TKA).

Patients who had undergone revision surgery for reasons other than infection and had been considered to be uninfected, but had had positive cultures within 5-days of surgery were identified. A thorough chart review was performed to determine their clinical outcome, and if they had undergone treatment for infection. True positive cases were defined as patients who subsequently developed PPI at 2-year follow-up and underwent treatment with intravenous antibiotics according to the infectious diseases specialist. Of the 704 arthroplasties that made up the aseptic cohort, 18 THAs and 3 TKAs fulfilled the above criteria and were considered to be truly infected, and they were therefore added to the septic group. Our final cohort consisted of 683 uninfected arthroplasties (401 THA, 282 TKA) that were revised for mechanical failure, and 321 (150 THA, 171 TKA) infected joints that underwent revision for PPI. The aseptic group consisted of 161 females and 160 males with an average age of 66 (25–97) years. The most common etiology of mechanical failure of TKA and THA was aseptic loosening of the components.

Resection arthroplasty with delayed reimplantation was done in 180 infected arthroplasties (117 TKA, 63 THA), while irrigation and debridement and one-stage reimplantation were performed in the remaining 120 cases. Of the additional 21 patients who were considered to be true positives, 4 patients (2 THA, 2 TKA) required additional surgical procedures to control the underlying infection, while the remaining 17 patients were successfully treated with 6 weeks of intravenous antibiotics without the need for further surgical intervention. The septic group consisted of 160 females and 161 males with an average age of 68 (23–88) years. The most common organisms encountered in the infected THA and TKA subgroups included methicillin-resistant *Staphylococcus aureus* (MRSA), methicillin-sensitive *Staphylococcus aureus* (MSSA), methicillin-resistant *Staphylococcus epidermidis* (MRSE), methicillin-sensitive *Staphylococcus epidermidis* (MSSE), *Streptococcus species,* Gram-negative species, and *Enterococcus faecalis* group D. An organism could not be isolated in 10% of infected TKAs and 4% of infected THAs.

The intraoperative specimens were obtained from representative sites including the joint capsule, synovium, and/or periprosthetic membrane or bone after the components were removed. Gram staining was performed at our institution by certified laboratory technicians using the standard method for Gram stain. This test has the capacity to detect Gram-positive organisms and Gram-negative organisms using the counterstain, while polymorphonuclear cells (PMNs) present in the specimens can also be visualized. PMNs were identified by their discrete lobular nuclear pattern, while cells with ill-defined cellular and/or nuclear borders secondary to the heat fixation of the Gram staining process were not included in the counts.

The specimen was first inspected by the laboratory technician on low-power field (20× magnification) to locate representative areas that were then scrutinized under oil immersion high-power field (HPF) with an area under the microscope of 2.6 × 10^4^ µm^2^. The amount of PMNs present in the specimens was classified according to the following scheme: many (> 5 PMNs per HPF), moderate (1–5 PMNs per HPF), and few (1 PMN per 2–10 HPFs). A Gram smear was defined as positive if “many” neutrophils or an organism was observed. The result of Gram stain was compared to the general diagnosis as determined by the criteria listed above. The above classification scheme is based on previous work by Chimento et al. (1995), who used a threshold of > 10 PMNs per HPF to diagnose infection and achieved a sensitivity and specificity of 18.8% and 95.6%, respectively. We reduced the cutoff value to > 5 PMNs per HPF in order to improve the sensitivity and negative predictive value of the test.

### Statistics

The estimated sensitivity, specificity, and predictive values were calculated for each diagnostic arm of Gram staining (presence of neutrophils or organisms), and the 95% confidence intervals (CIs) are reported for both TKA and THA separately. Parallel combination testing requires any one or both of the tests to be positive to diagnose infection, and therefore improves the sensitivity of the combined tests and their ability to rule out infection. On the other hand, series combination testing requires both tests to be positive (and not just either one) to reach a diagnosis of PPI. This improves the specificity of the combination and allows the surgeon to confirm PPI more accurately (Griner et al. 1981). All statistical analyses were performed using SAS version 9.1 (SAS Institute, Cary, NC).

## Results

Gram staining failed to diagnose PPI in 80 infected TKAs (47%) and 103 infected THAs (69%) when using the presence of “many” neutrophils as criteria for infection. Similarly, a stained organism was observed in only 30% of TKAs and 31% of THAs with confirmed PPI. Although the sensitivity of Gram staining ranged from 30% to 53%, the negative predictive value was slightly higher (70–79%) (Table 1). The specificity of the two diagnostic arms of Gram staining was 99–100% in both types of total joint arthroplasty. Of 683 uninfected arthroplasties, only 1 false positive reading was shown, in which an organism was observed in specimens from an infected TKA. However, a greater number of false positives occurred in both THA (6 patients) and TKA (4 patients) when using ‘many’ neutrophils as the criterion for diagnosing infection.

**Table 1.** The sensitivity, specificity, positive predictive value (PPV) and negative predictive value (NPV) of the 2 diagnostic arms of Gram staining

Diagnostic arm	Sensitivity (%)	Specificity (%)	PPV (%)	NPV (%)
*THA*				
Presence of organisms	31 (23–38)	100 (99–100)	100 (92–100)	79 (76–83)
Presence of “many” PMNs	31 (24–39)	99 (97–99)	89 (80–97)	79 (75–83)
*TKA*				
Presence of organisms	30 (24–37)	100 (99–100)	98 (94–100)	70 (66–75)
Presence of “many” PMNs	53 (46–61)	99 (97–99)	96 (92–99)	78 (73–82)

When either an organism was detected on the Gram smear or more than 5 PMNs per HPF were present in a sample, the sensitivity and negative predictive value of this combination in THA were 43% and 82%, respectively. Similar improved sensitivity and negative predictive value were obtained for TKA when using the same methodology of combining tests (Table 2). However, the false negative rate for both types of joints remained appreciable (36–57%). 9 false positive cases (5 THA, 4 TKA) were observed. The specificity, however, was 99% for both types of joint surgery. On the other hand, combining both diagnostic arms of Gram staining in series, which requires that both tests be positive in order to diagnose an infection, can achieve absolute specificity (100%).

**Table 2.** Combinations of the 2 arms of Gram staining were performed in series and in parallel for both types of joints. PPV: positive predictive value; NPV: negative predictive value

Combinations	Sensitivity (%)	Specificity (%)	PPV (%)	NPV (%)
*THA*				
Series	18 (12–24)	100 (99–100)	100 (87–100)	77 (73–80)
Parallel	43 (35–51)	99 (97–99)	93 (84–98)	82 (79–86)
*TKA*				
Series	21 (15–27)	100 (99–100)	100 (90–100)	68 (63–72)
Parallel	64 (57–72)	99 (97–99)	97 (91–99)	82 (77–86)

The cohorts of TKA and THA were merged and then stratified according to the total number of specimens obtained intraoperatively, to determine the effects of increasing sample size on the diagnostic value of the Gram stain. The 4 subgroups consisted of infected and uninfected patients who had 1, 2, 3, or 4 intraoperative samples taken. The specificity and positive predictive value of the neutrophil dimension of the test ranged from 97% to 100% and 90% to 100%, respectively, among the four groups. Similarly, the presence of an organism had elevated specificity and positive predictive value for all 4 subgroups (Table 3). The sensitivity of the neutrophil arm improved with increasing number of specimens, and reached a peak of 50%. However, the negative predictive value steadily declined with a greater number of samples and reached a nadir of 52%. In contrast, the sensitivity of visualizing a Gram-stained organism did not show major improvement with increasing number of samples (Table 3). The negative predictive value of this diagnostic arm steadily dropped with greater numbers of samples, from 89% for one sample to 44% for 4 samples.

**Table 3.** Sensitivity, specificity, positive predictive value (PPV) and negative predictive value (NPV) for increasing specimen numbers obtained intraoperatively

Sample No. Test	Sensitivity (%)	Specificity (%)	PPV (%)	NPV (%)
*1*				
Presence of organisms	32 (20–44)	100 (99–100)	95 (85–100)	89 (86–92)
Presence of “many” PMNs	29 (17–40)	99 (98–99)	90 (76–100)	89 (85–92)
*2*				
Presence of organisms	28 (20–35)	100 (98–100)	100 (91–100)	72 (67–76)
Presence of “many” PMNs	43 (35–51)	97 (95–99)	90 (82–97)	76 (75–80)
*3*				
Presence of organisms	29 (20–40)	100 (95–100)	100 (86–100)	55 (46–63)
Presence of “many” PMNs	47 (36–58)	97 (93–100)	95 (89–100)	61 (51–70)
*4*				
Presence of organisms	31 (50–84)	100 (80–100)	100 (69–100)	44 (28–61)
Presence of “many” PMNs	50 (33–67)	100 (80–100)	100 (79–100)	52 (34–68)

## Discussion

Feldman et al. (1995) found that Gram stain was able to properly diagnose infection in only 2 of 10 infected arthroplasties. Pandey et al. (2000) reported similar sensitivity (21.5%) in a retrospective review of 602 revision THAs. Similarly, Spangehl et al. (1999b) conducted a prospective evaluation of 202 total hip revisions in which the sensitivity and negative predictive value of the Gram stain were 19% and 89% respectively, which led the authors to abandon this test in favor of frozen sections.

The presence of neutrophils on Gram-stained vaginal swabs has been associated with preterm birth (Ramsey et al. 2005). Currently, male urethral infections are confirmed by the presence of ≥ 5 PMNs per HPF in urethral secretions (Geisler et al. 2005), while the presence of Gram-stained neutrophils in prostatic secretions is indicative of chronic prostatitis (Krieger et al. 2003). Pathologists frequently use the histological criteria set forth by Mirra et al. (1976), who defined PPI as the presence of 5 or more neutrophils per high-power field in more than 5 fields in permanent histological sections. In our study, we applied similar criteria to the results of Gram staining but with different neutrophil thresholds in order to improve the sensitivity and negative predictive value.

Visualization of more than 5 PMNs per HPF achieved disappointingly low sensitivity (31–53%) and a moderate negative predictive value (78–79%) for both THA and TKA. A slightly lower sensitivity (19%) was reported by Chimento et al. (1995), who used 10 neutrophils per high-power field to confirm PPI. In our cohort, the specificity and positive predictive value of the two subdivisions of Gram staining approached absolute levels in both THA and TKA. The sensitivity and negative predictive value of the Gram stain improved in THA and TKA when both diagnostic arms of the Gram stain were combined in series. However, the false negative rate of the combination is still too high to safely rule out or exclude PPI, even when both branches of Gram staining are negative. Furthermore, combining both tests when they are positive adds little to the specificity and positive predictive value, possibly because the individual diagnostic arms have near-absolute specificities in the first place.

We hypothesized that performing Gram staining on a greater number of specimens might improve the ability of the test to exclude infection. The false negative rate when using “many” neutrophils as criterion for infection decreased from 71% for 1 sample under analysis to 50% for 4 samples. We did not detect such improvement when using the presence of an organism to diagnose infection, while the sensitivity remained approximately 30% across the 4 sample subgroups. We conclude that obtaining multiple intraoperative samples does not increase the sensitivity, nor alleviate the problem of high false negative rates.

One caveat must be kept in mind when assessing neutrophils on a Gram smear. The Gram stain technique is harsh on neutrophil cells, due to the heat fixation process that affects the integrity of the cell. To date, there are no special processing techniques that can preserve the integrity of human leukocytes when performing Gram staining. Hence, alternative staining methods that are friendlier to neutrophils (including Giemsa and Wright’s stain) should be assessed in a prospective study. The efficacy of other stains—including acridine orange—in staining bacteria should also be addressed.
